# Inverted supracondylar femoral dynamic compression plates for revision of construct failure following tibial cranial closing wedge ostectomy in three dogs

**DOI:** 10.1111/jsap.13817

**Published:** 2024-12-26

**Authors:** S. Wells, J. Winter, M. Pead, R. Meeson

**Affiliations:** ^1^ The Queen Mother Hospital for Animals Royal Veterinary College Hatfield UK

## Abstract

Construct failure is a rare complication of tibial cranial closing wedge ostectomy for the management of cranial cruciate ligament disease. Construct failure can present significant challenges for reconstruction. This case report describes three dogs with construct failure which were successfully revised by the repurposing of an inverted femoral supracondylar femoral plate as part of the revision. Data were obtained from the medical records and telephone update. The use of an inverted supracondylar plate in the proximal tibia generally had good outcomes for the management of bone implant failure following cranial closing wedge ostectomy and offers a hitherto undescribed technique using a low‐cost and accessible implant for repair.

## BACKGROUND

The first tibial osteotomy described to address a ruptured cranial cruciate ligament in the dog was the cranial closing wedge ostectomy (CCWO) (Slocum & Devine, 1984) and has been largely superseded by the tibial plateau levelling osteotomy (TPLO) (Slocum & Slocum, 1993; von Pfeil et al., 2018). However, the CCWO purportedly remains advantageous in small dogs, dogs with a narrow proximal tibia, and dogs with an excessive tibial plateau angle, and does not require specialised crescentic saws (Oxley, 2021). The reported complication rates for CCWO vary between 9% and 31.7% and construct failure through breakage of the implants or bone failure/fracture can occur in up to 14% of dogs (Brown & Corr, 2007; Kuan et al., 2009; Oxley et al., 2013). Revision surgery can be challenging due to the limited bone stock due to previous implant holes and fracture/comminution of the proximal fragment, not to mention the large mechanical forces acting upon the fragment which are created by the quadriceps combined insertion onto the margo cranialis tibiae from the patellar tendon. Documented repair techniques include pin and tension band wire (Kuan et al., 2009) and external skeletal fixation (Campbell et al., 2016). The three cases presented herein were revised using a novel application of an anatomically designed plate intended for supracondylar femoral fractures, yet applied to the tibia inverted. This achieved a screw hole configuration away from the original screw holes and provided a long diaphyseal component to bridge across the previous implants' diaphyseal holes.

## CASE HISTORIES

The cases presented herein, represent all the cases of tibial closing wedge ostectomy where a revision was performed using a novel application of an inverted femoral supracondylar dynamic compression plate.

### Case 1

**FIG 1:**
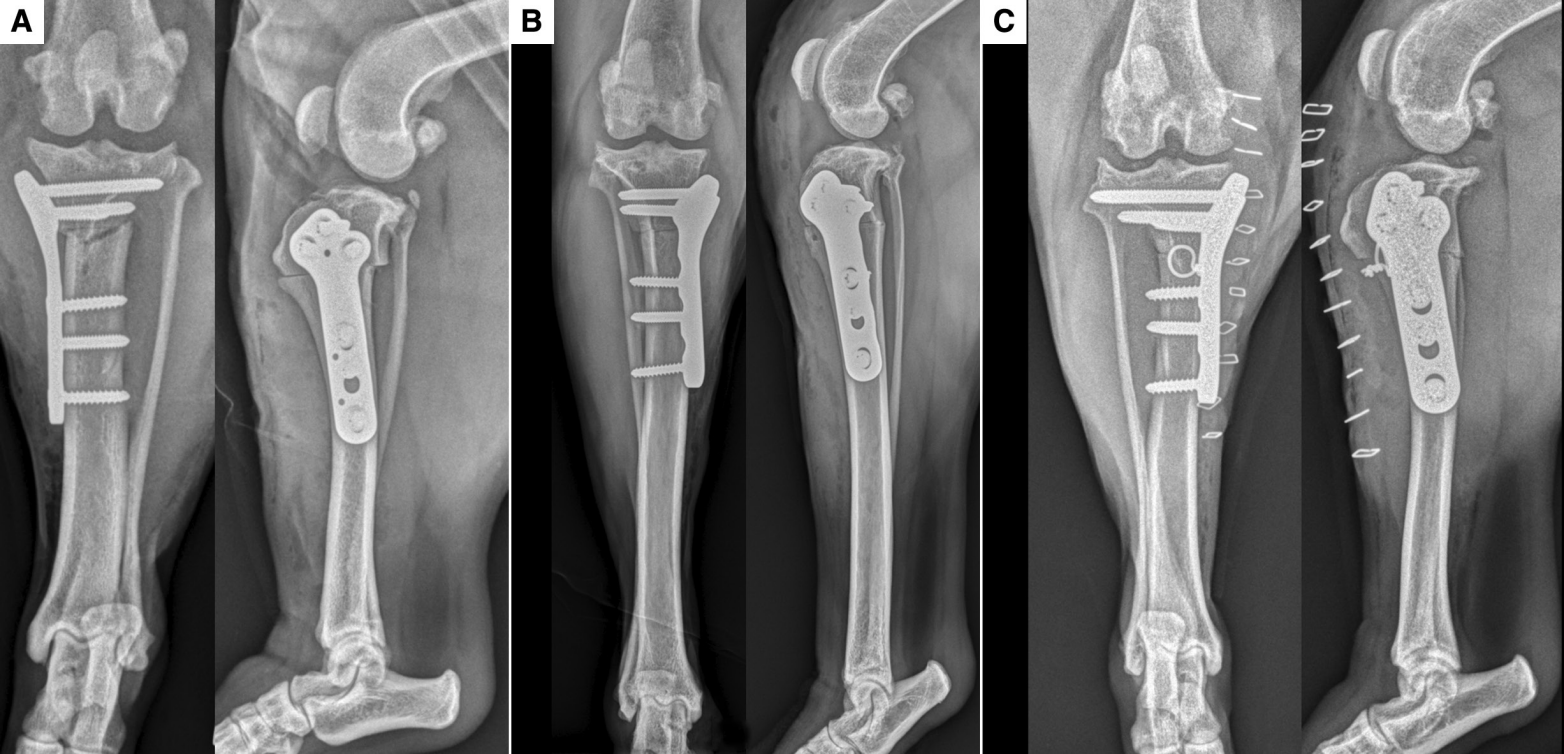
Immediate post‐operative radiographs following CCWO for cases 1 (A), 2 (B) and 3 (C).

**FIG 2:**
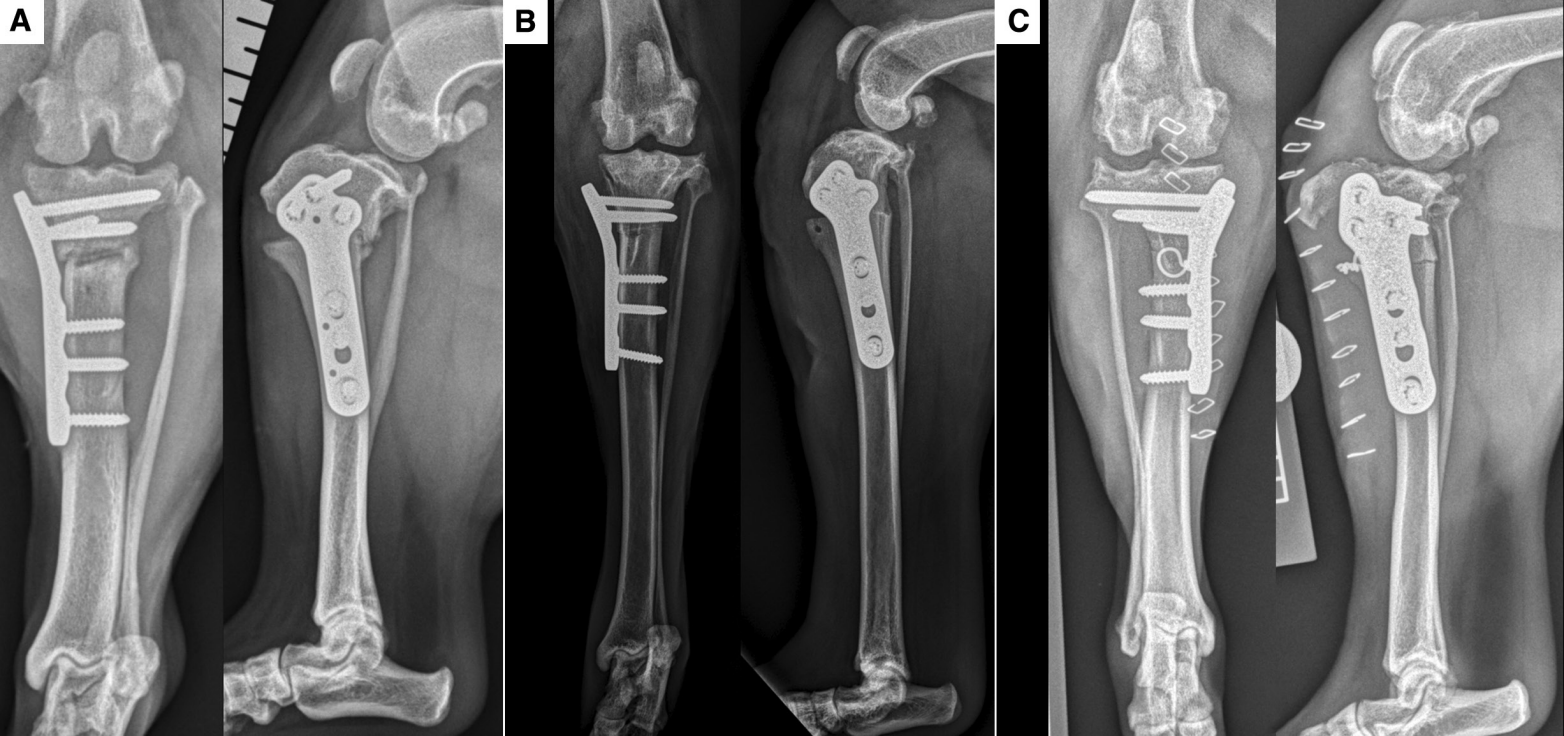
Follow‐up radiographs demonstrating construct failure for cases 1 (A), 2 (B) and 3 (C).

A 1‐year, 11‐month‐old female neutered cross‐breed presented for CCLD of the left hindlimb and underwent an isosceles CCWO (Oxley et al., 2013) with a 2.7‐mm locking TPLO plate (Depuy Synthes, Solothurn), without a cranial loop wire (Fig 1A). Seven weeks following surgery, the dog was represented with a worsening lameness (7/10) and radiographs showed proximal fragment instability due to proximal screw loosening and bone fracture (Fig 2A).

**FIG 3:**
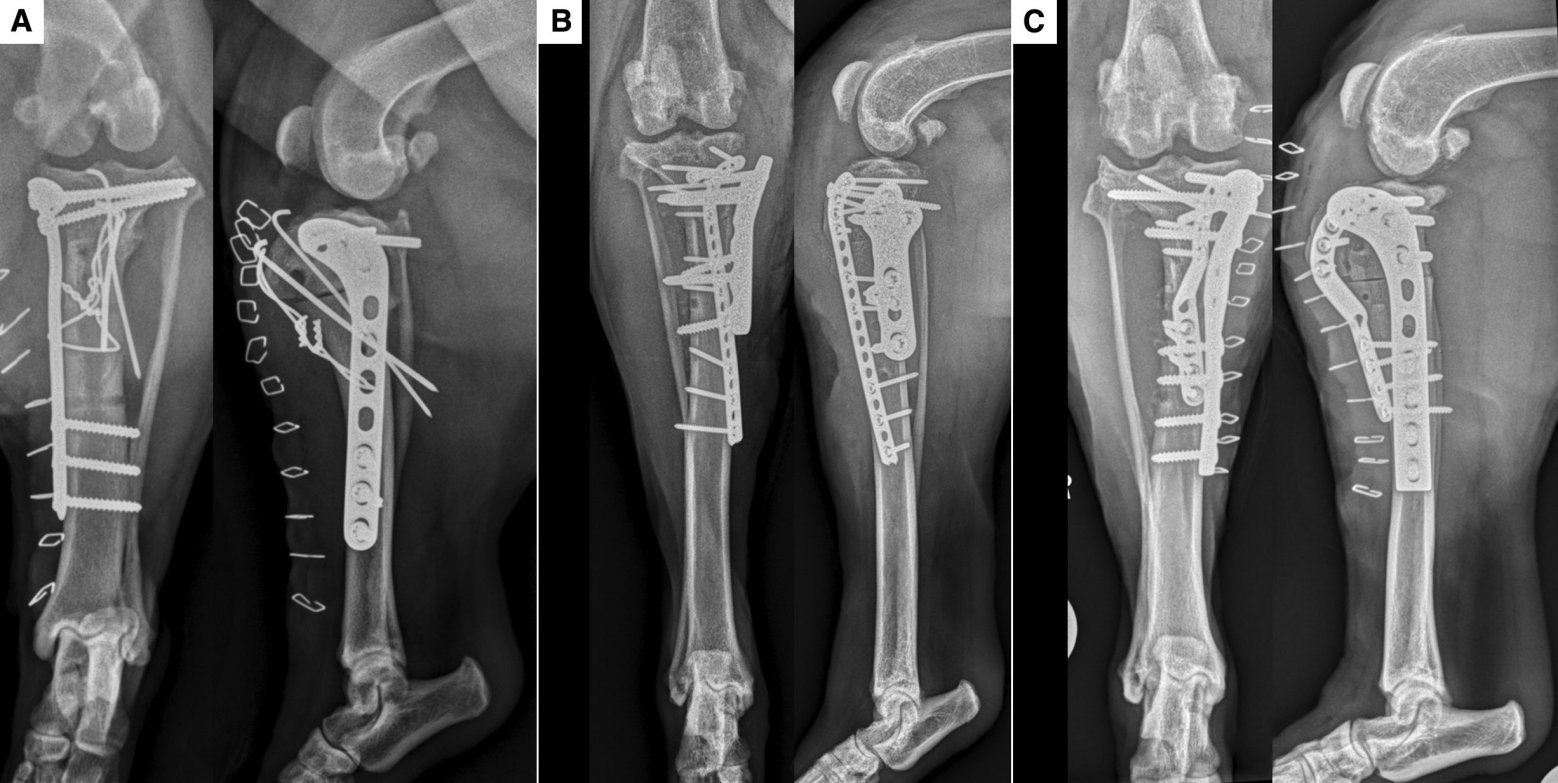
Immediate post‐operative radiographs following revision surgery with inverted femoral supracondylar plates for cases 1 (A), 2 (B) and 3 (C). The k‐wires in (A) were noted to be excessively long but there was concern that revising them at that stage may destabilise the fragile fragment, and so it was opted to monitor for discomfort.

The surgery was revised through a medial approach to the proximal tibia. After implant removal, the proximal screw holes were found to be widened and a delayed‐union present. The two fragments were debrided, aligned and stabilised with a 1.2‐mm Kirschner wire. A 10‐hole 2.4‐mm left‐sided supracondylar dynamic compression plate (Veterinary Instrumentation, Sheffield) was contoured to the proximal tibia and applied along the caudal margin of the medial tibia. Three 2.4‐mm bi‐cortical screws were placed in either fragment. A second 1.0‐mm Kirschner wire was placed alongside the first with a 0.8‐mm tension band wire. Demineralised bone matrix (DBM) was packed between the osteotomy edges prior to reduction and then further added immediately prior to closure. Post‐operative radiographs confirmed good reduction and appropriate implant placement (Fig 3A). The K‐wires were noted to be excessively long but there was concern that revising them at that stage may destabilise the fragile fragment. It was opted to monitor for discomfort and no discomfort associated with the pins was found.

**FIG 4:**
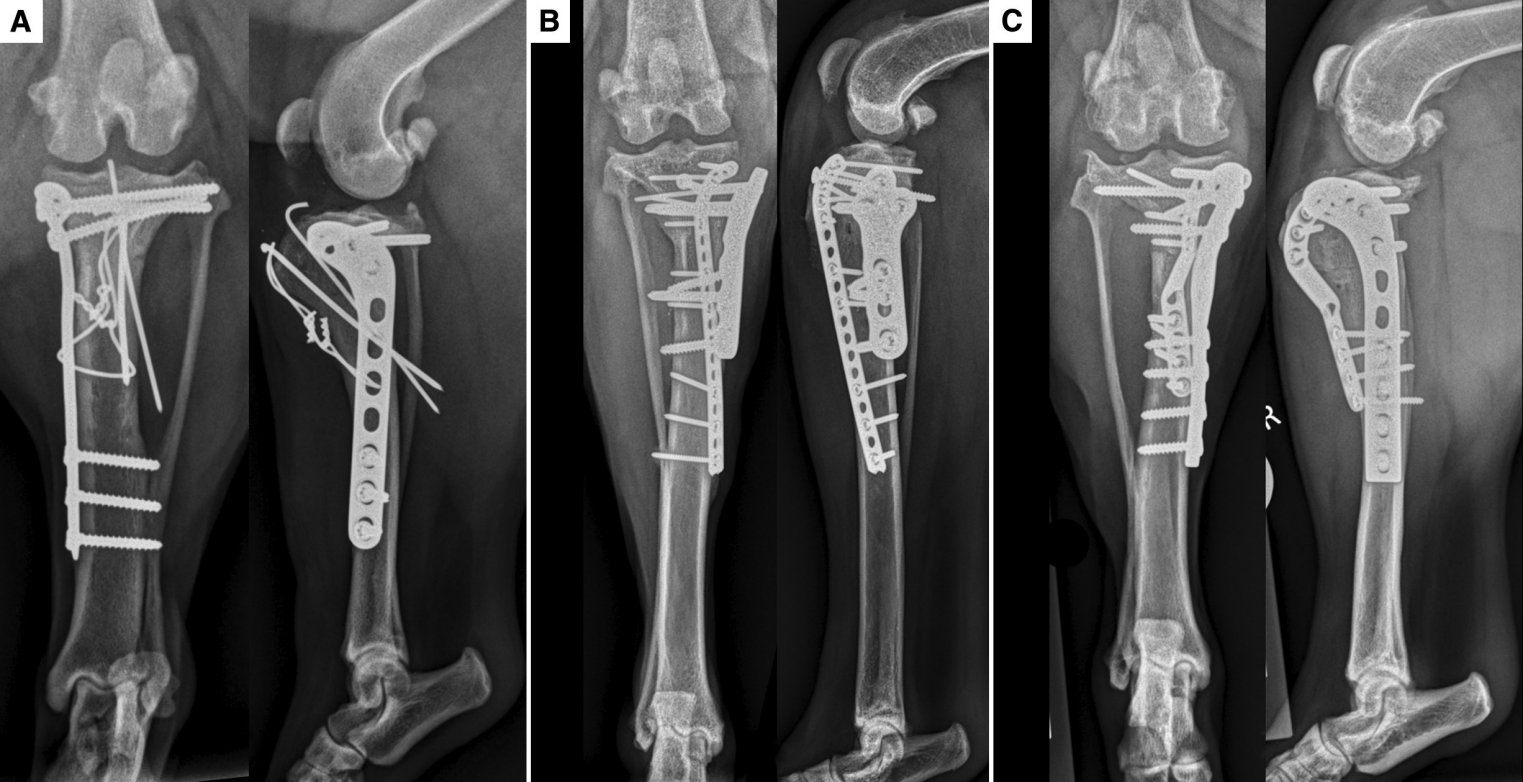
Follow‐up radiographs following revision surgery demonstrating radiographic signs of healing for cases 1 (A), 2 (B) and 3 (C).

Final re‐check at 14 weeks following revision surgery showed a mild left hindlimb lameness (1/10) and mild left hindlimb muscle atrophy. Radiographs showed stable implants with complete osseus union (Fig 4A). Telephone follow‐up with the canine brief pain inventory score (CBPI) at 1 year revealed an intermittent lameness on a weekly basis and oral meloxicam was administered when required. The owner‐assessed outcome was graded good.

### Case 2

A 6‐year, 2‐month‐old female neutered Labrador presented for CCLD of the right hindlimb and underwent an isosceles CCWO (Oxley et al., 2013) with a 3.5‐mm locking TPLO plate (Depuy Synthes, Solothurn) and a 1.0‐mm interfragmentary wire. During surgery the wire dissected through the proximal segment into the osteotomy and so was removed (Fig 1B).

The dog failed to weight bear as expected following surgery, and radiographs at 5 days revealed proximal segment instability with a caudal stepped fissure propagating from the osteotomy proximally (Fig 2B). Revision surgery was performed from a medial approach to the proximal tibia. A fracture had propagated from the osteotomy surface through the bone defect from the aborted wire application and on to the cranial two screw holes of the plate. After implant removal, the fracture line was reduced and stabilised with a 2.7‐mm lag screw from cranial to caudal. A left‐sided 2.0‐mm supracondylar dynamic compression plate (Veterinary Instrumentation, Sheffield) was contoured and placed inverted on the cranial aspect of the medial tibia with five bi‐cortical screws in both proximal and distal fragments. A 3.5‐mm “Small” TPLO plate (Depuy Synthes, Solothurn) was also applied to the caudomedial aspect of the tibia with three bi‐cortical locking screws proximally and two bi‐cortical screws and one locking screw distally. Post‐operative radiographs demonstrated good reduction and appropriate implant placement (Fig 3B).

*Re*‐examination at 8 weeks showed a 2/10 lameness with moderate peri‐articular thickening and a mild stifle effusion. Radiographs showed stable implants and progressive healing with the osteotomy and fracture line no longer distinguishable (Fig 4B). Telephone follow‐up with CBPI at 4 years identified no lameness or discomfort. The owner‐assessed outcome was graded excellent.

### Case 3

A 5‐year, 11‐month‐old female neutered cocker spaniel presented for CCLD affecting the right hindlimb and underwent an isosceles CCWO (Oxley et al., 2013) with a 3.5‐mm “small” locking TPLO plate (Depuy Synthes, Solothurn) and a 1.0‐mm interfragmentary wire (Fig 1C).

The dog represented 2 weeks post‐operatively for acute onset right hindlimb lameness (8/10). Radiographs showed a fracture of the proximal fragment extending from the interfragmentary wire hole and through the cranial screw holes (Fig 2C). Revision surgery was performed from a medial approach to the proximal tibia, which revealed a fracture line between the most cranial and proximal screw holes. The proximal and distal fragments were then stabilised with a 1.0‐mm Kirschner wire and the TPLO plate removed. A right‐sided 2.7‐mm DCP supracondylar plate (Veterinary Instrumentation, Sheffield) was contoured and placed inverted along the caudal aspect of the medial surface of the tibia and secured with four 2.7‐mm bi‐cortical screws in the distal and caudo‐proximal fragments. The tibial crest fragment was reduced and stabilised with an additional left‐sided inverted 2.0‐mm supracondylar plate (Veterinary Instrumentation, Sheffield) cranially on the medial tibial surface with three 2.0‐mm bi‐cortical screws in the proximal fragment and four 2.0‐mm bi‐cortical screws in the distal fragment. Post‐operative radiographs showed good reduction and appropriate implant placement (Fig 3C).

*Re*‐examination at 8 weeks demonstrated a 2/10 lameness. Radiographs showed stable implants and progressive bone healing with the osteotomy and fracture lines indistinguishable (Fig 4C). Telephone follow‐up with CBPI at 3 years confirmed no lameness or discomfort and ability to exercise was unimpaired. The owner‐assessed outcome was graded excellent.

## DISCUSSION

This series describes three cases of construct failure following CCWO which were successfully revised using combinations of inverted femoral supracondylar dynamic compression plates. CCWO failures are challenging to manage due to the small proximal fragment alongside the presence of screw holes from the previously applied TPLO plate. If the pattern of failure precludes continued use of the TPLO plate screw holes, or fracture results in splitting of the proximal fragment, an alternate strategy is required to maximise the remaining bone stock and achieve a rigid fixation to enable successful healing. The femoral supracondylar plate possesses several features which are advantageous in this situation. It is longer than a TPLO plate which enables bridging of the previous diaphyseal screw holes. The pattern of screw distribution is also different to TPLO plates, meaning that the previous screw holes can be avoided. The plate is readily available, inexpensive, comes in a range of sizes, is familiar to orthopaedic surgeons and requires no special equipment. A “left‐handed” or “right‐handed” plate can be selected and preferentially applied in a more caudal or cranial position along the medial tibia, increasing flexibility in location of screw holes, or indeed one of each plate could be used. The only difficulty can be achieving appropriate contouring in this position on the tibia.

It has been demonstrated that locking implants reduce fragment movement in TPLO and locking screws have been shown to provide mechanical advantages over cortical screws, improving construct stability and avoiding primary loss of reduction and reducing subsequent rock‐back (Conkling et al., 2010; Leitner et al., 2008). However, although the femoral supracondylar plate does not have locking screws, the cortical (or cancellous) screws placed allow for variable orientation to avoid fissures or engage parts of the bone which would not be possible with a fixed‐angle locking system. One case allowed for the placement of a locking TPLO plate in addition to the supracondylar plate, potentially giving the combined benefit of increased fragment stability from the locking plate, with the beneficial screw spread and avoidance of holes and fissures from the supracondylar plate.

Two cases experienced fractures of the proximal fragment through the inter‐fragmentary wire hole. It may be that the additional cranial hole, or its proximity to the plate holes potentiated the development of a fracture. An alternative to an inter‐fragmentary wire is a cranial pin and tension band wire which is biomechanically beneficial for stabilising fragments with significant avulsive forces. This was implemented in the revision of case one. In cases two and three, the proximal segment was better served by a plate acting as a tension band and simultaneously reconstructing the multi‐segmental proximal fragment.

Case 1 had DBM applied at the time of the revision due to the chronicity of the instability. Although DBM is only osteoinductive with no osteogenic potential, there is evidence that it is equivalent to cancellous autogenous bone graft in canine arthrodesis (Hoffer et al., 2008).

Cases 2 and 3 went on to achieve full function as per Cook et al. (2010) at final telephone follow‐up. Case one achieved acceptable function and the owners described an intermittent lameness. Due to the lack of clinical follow‐up, we cannot comment on the cause of intermittent lameness, however general stifle disease and secondary osteoarthritis could also explain the clinical findings. Ultimately all revisions healed, and no further construct failure was seen during an appropriate follow‐up time period.

In summary, this case series provides the first description of the advantages of the application of inverted distal femoral supracondylar plates being used in the proximal tibia for bone‐implant failure after CWO. All three cases went on to heal without further mechanical failure and outcomes were generally good.

Ethical approval was gained from the Royal Veterinary College (RVC) Social Science Research Ethical Review Board (SSERB) for the use of retrospective date and the collection of long‐term follow‐up data.

## Author contributions

**S. Wells:** Data curation (equal); project administration (equal); writing – original draft (equal). **J. Winter:** Conceptualization (equal); methodology (equal); project administration (equal); supervision (equal); writing – original draft (equal); writing – review and editing (equal). **M. Pead:** Conceptualization (equal); methodology (equal); writing – review and editing (equal). **R. Meeson:** Conceptualization (equal); methodology (equal); supervision (equal); writing – review and editing (equal).

## Conflict of interest

None of the authors of this article has a financial or personal relationship with other people or organisations that could inappropriately influence or bias the content of the paper.

## Data Availability

Data available on request from the authors.
